# Optimizing *Phaeodactylum tricornutum* cultivation: integrated strategies for enhancing biomass, lipid, and fucoxanthin production

**DOI:** 10.1186/s13068-024-02602-5

**Published:** 2025-01-18

**Authors:** Mostafa E. Elshobary, Walaa A. Abo-Shanab, Stephan S. W. Ende, Mohammed Alquraishi, Rania A. El-Shenody

**Affiliations:** 1 Botany and Microbiology Department, Faculty of Science, Tanta University, Tanta, 31527 Egypt; 2 Aquaculture Research, Alfred Wegener Institute (AWI) – Helmholtz Centre for Polar and Marine Research, Am Handelshafen, 27570 Bremerhaven, Germany; 3 Department of Community Health Sciences, College of Applied Medical Sciences, King Saud University, 11433 Riyadh, Saudi Arabia

**Keywords:** Diatom, Fucoxanthin, Biodiesel properties, Lipid optimization, Fatty acid profile, Mixotrophic cultivation

## Abstract

**Background:**

*Phaeodactylum tricornutum* is a versatile marine microalga renowned for its high-value metabolite production, including omega-3 fatty acids and fucoxanthin, with emerging potential for integrated biorefinery approaches that encompass biofuel and bioproduct generation. Therefore, in this study we aimed to optimize the cultivation conditions for boosting biomass, lipid, and fucoxanthin production in *P. tricornutum*, focusing on the impacts of different nutrient ratios (nitrogen, phosphorus, silicate), glycerol supplementation, and light regimes.

**Results:**

Optimized medium (− 50%N%, + 50% P, Zero-Si, 2 g glycerol) under low-intensity blue light (100 μmol m⁻^2^ s⁻^1^) improved biomass to 1.6 g L⁻^1^, with lipid productivity reaching 539.25 mg g⁻^1^, while fucoxanthin increased to 20.44 mg g^−1^. Total saturated fatty acid (ΣSFA) content in the optimized culture increased approximately 2.4-fold compared to the control F/2 medium. This change in fatty acid composition led to improved biodiesel properties, including a higher cetane number (59.18 vs. 56.04) and lower iodine value (53.96 vs 88.99 g I_2_/100 g oil). The optimized conditions also altered the biodiesel characteristics, such as kinematic viscosity, cloud point, and higher heating value.

**Conclusion:**

Our optimization approach reveals the significant potential of *P. tricornutum* as a versatile microbial platform for biomass, lipid, and fucoxanthin production. The tailored cultivation strategy successfully enhanced biomass and lipid accumulation, with notable improvements in biodiesel properties through strategic nutrient and light regime manipulation. These findings demonstrate the critical role of precise cultivation conditions in optimizing microalgal metabolic performance for biotechnological applications.

**Supplementary Information:**

The online version contains supplementary material available at 10.1186/s13068-024-02602-5.

## Background

The unsustainable nature of conventional fossil fuels has become increasingly apparent due to their significant contribution to climate change and greenhouse gas emissions [1]. In response, biofuels derived from microalgal sources have emerged as a promising research avenue, offering the potential for reduced carbon emissions and efficient solar energy conversion [2]. Lipid-based biofuels have attracted scientific interest due to their potential for higher energy density and broader potential applications [1, 3].

Microalgae present unique advantages as potential renewable biofuel feedstocks, demonstrating superior light utilization and growth characteristics compared to terrestrial plants. With a light-to-biomass conversion efficiency of 6–8%, significantly higher than the 1–2% observed in land plants, microalgae represent an intriguing experimental platform for bioenergy research [4]. Their cultivation offers the potential to avoid competing with food crop agriculture by utilizing marine and freshwater environments [5, 6]. These photosynthetic microorganisms, widely distributed in oceans and critical to marine primary productivity, accumulate energy-rich lipids as triacylglycerols in cellular oil bodies [7]. Microalgal biofuels represent a promising area of research for sustainable energy production, with ongoing efforts to overcome technical and economic challenges in large-scale implementation. Research continues to explore their potential for producing various biofuel precursors, including experimental approaches to biogas, biohydrogen, biobutanol, and bioethanol production [4, 8]. Recent studies have explored the effects of nutrient combinations on diatom growth and productivity. Bhattacharjya et al. [9] found that high eutrophic nutrient media increased biomass productivity and cellular components in marine diatoms. Li et al. [10] demonstrated that micronutrient addition and adjusted N:P ratios significantly enhanced diatom growth and nutrient removal in aquaculture wastewater. Giri et al. [11] reviewed the importance of nitrogen, phosphorus, iron, and silica for diatom growth, noting their potential toxicity at high concentrations.

Diatoms are a unique class of microalgae distinguished by their exceptional metabolic versatility, particularly in the production of high-value compounds including lipids and carotenoids including *Phaeodactylum tricornutum, Cyclotella cryptica, Isochrysis aff. galbana, Odontella aurita, and Chaetoceros calcitrans* [12]. Their most distinctive characteristic is the abundant production of fucoxanthin, a xanthophyll carotenoid that gives them their characteristic golden-brown color and demonstrates remarkable therapeutic properties, including anti-obesity, anti-diabetic, anti-cancer, and anti-inflammatory effects [13]. Additionally, diatoms excel in producing omega-3 fatty acids and accumulating energy-rich lipids, predominantly triacylglycerols (TAGs), making them attractive candidates for both nutraceutical applications and biodiesel synthesis [13, 14].

Among marine diatoms, *Phaeodactylum tricornutum* stands out as a vital model organism for diverse scientific research. This versatile microorganism exhibits remarkable metabolic flexibility through photoautotrophic and mixotrophic growth and impressive lipid accumulation capacity (20–60% of dry weight) and fucoxanthin content reaches 2.5% under stress conditions [15, 16]. Its ability to accumulate triacylglycerols (TAGs; 20–30% of biomass) and synthesize valuable omega-3 fatty acids, particularly eicosapentaenoic acid (EPA), positions it as a promising candidate for both biodiesel production and nutraceutical applications [17].

Nutrient strategies have proven particularly effective in modulating lipid and fucoxanthin accumulation in diatoms, with a specific focus on nitrate, phosphate, and silicate availability [10]. Nitrogen limitation emerges as a critical strategy in diatom lipid production, fundamentally redirecting cellular carbon flux from protein synthesis to lipid biosynthesis [18]. However, this approach presents a nuanced challenge, as it frequently results in reduced biomass yield [19]. The delicate balance between lipid accumulation and biomass maintenance represents a key research focus in diatom biotechnology. Phosphorus and silicate manipulations offer additional unique opportunities in diatom cultivation. Phosphorus stress can trigger substantial metabolic shifts, potentially increasing both growth rate and lipid accumulation [20]. Silicate manipulation, intrinsic to diatoms due to their silica cell walls, presents a distinctive avenue for lipid induction. Investigations have shown that strategic silicon deprivation can stimulate lipid accumulation without significantly compromising growth rates in certain diatom species [21]. Moreover, the degree of nutrient manipulation is crucial, with studies showing that manipulating nutrients can optimize biomass and lipid productivity [22]. Carbon sources such as glycerol have emerged as promising substrates for enhancing biomass, pigments, and lipid production in diatoms, offering a potential route to improve their viability as a biofuel feedstock and nutraceutical applications [23, 24]. Glycerol supplementation can enhance diatom growth such as *P. tricornutum* under various stress conditions, including nitrogen limitation, potentially offering a strategy to optimize both biomass and proximate productivity [25, 26].

Aside from nutrient manipulation, light is essential for developing and metabolizing diatoms including *P. tricornutum*. The intensity, quality, and duration of light contact significantly influence these organisms’ photosynthetic efficiency, biomass production, and cellular composition [27]. In particular, diatoms have evolved sophisticated mechanisms to adapt to varying light conditions, allowing them to thrive across various light intensities [28, 29]. Recent investigations have shown that manipulating light conditions can effectively improve lipid accumulation and photosynthetic pigments in diatoms [30]. Understanding the complex interplay between light intensity and diatom physiology is crucial for optimizing cultivation conditions and maximizing biomass and lipid productivity in microalgae [31].

While previous studies have explored individual factors affecting *P. tricornutum* cultivation, our study presents several novel aspects. First, we investigate the synergistic effects of precise nutrient ratios (nitrogen, phosphorus, and silicate) combined with glycerol supplementation and specific light regimes on a newly isolated Mediterranean strain. Second, we employ a holistic optimization approach that simultaneously targets three valuable products: biomass, lipids, and fucoxanthin. Third, our study provides a comprehensive analysis of biodiesel properties resulting from the optimized cultivation conditions. This integrated strategy, applied to a locally isolated strain, offers new insights into strain-specific responses to environmental manipulation and presents a novel framework for optimizing multiple high-value products simultaneously. The results will advance our understanding of diatom metabolism under combined stress conditions and provide practical guidelines for developing efficient, multi-product biorefinery approaches using *P. tricornutum*.

## Material and methods

### Isolation, cultivation, and molecular identification

Water samples from Alexandria, Egypt (31◦12,035.900 N; 29◦52,058.400 E) were collected in 2022 using sterile glass bottles. Under controlled conditions, the samples were cultured in a modified F/2 medium [32] (supplemented materials). Cultivation was carried out in a Multitron II incubator (Infors-HT, Bottmingen, Switzerland) under continuous illumination at 100 μmol m⁻^2^ s⁻^1^ and orbital agitation (150 rpm) for 10 days. After growth was observed, the culture was spread on a solid medium containing 1.5% agar (Sigma; USA) and purified through repeated subculturing. Microscopic examination tentatively identified the isolate as *Phaeodactylum* sp. [33] using an Olympus BX51 light microscope (Olympus, Tokyo, Japan). The algae were preserved on agar slants at 4 °C for long-term storage.

To confirm the identity of *Phaeodactylum* sp., molecular identification was conducted using the universal 18S rRNA gene (CDMF (5ʹ GTC AGA GGT GAA ATT CTT GGA TTT A 3ʹ) and CDMR (5ʹ AAG GGC AGG GAC GTA ATC AACG 3ʹ) following standard protocols using the ABI PRISM® 3100 Genetic Analyzer (Micron–Corp. Korea). PCR amplifications were performed following the method of Elshobary et al. [29]. Genetic analysis involved comparing sequences using NCBI’s BLAST tool. MEGA11 software was used to align sequences and construct phylogenetic trees. The trees were built using the maximum likelihood (ML) method based on Tamura-Nei model [34], the neighbor-joining (NJ) algorithm [35] based on parameter distance (PD) [35, 36] and maximum parsimony (MP) based on tree bisection and reconnection (TBR) [36] branch swapping in MEGA 11 [37]. Significant bootstrap values (≥ 70%) are shown in the final tree. The node support was conducted using 1000 bootstrap resamplings [38].

### Optimization of biomass and lipid production

To enhance the production of biomass, lipids, and fucoxanthin in *P. tricornutum*, growth conditions were fine-tuned through laboratory experiments (supplemented materials). The nutrient trials used sodium nitrate for nitrogen, sodium dihydrogen phosphate for phosphorus, and sodium metasilicate nonahydrate for silicon. Fourteen experiments containing modified medium were carried out, and the 1st group was the control. Different concentrations of phosphorus, silicate and nitrogen concentration were prepared in optimized media as follows [nitrogen (N): Zero-N, − 50%N%, + 50%N; phosphorus (P): Zero-P, − 50%P, P + 50%P; silicate (Si): Si-Zero, − 50%Si, + 50%Si and glycerol concentration of (0.5, 1, 1.5, and 2 g L**⁻**^**1**^)]. The experiments were carried out in 500-mL Erlenmeyer flasks, each containing 350 mL of the designed medium (Table S1). The inoculation amount was 10% (v/v) of exponentially growing algal culture with an initial optical density (OD) of 0.1 at 750 nm. During the experiment, a temperature of 20 ± 1 °C at a continuous irradiance of 100 μmol m^−2^ s^−1^ was held constant. These flasks were aerated using a filtered ambient air pump to preserve algal homogeneity with the experimental media. F/2 medium was used as a control. The concentrations of nitrate, glycerol, and phosphorus were selected based on their role in promoting optimal growth and metabolite production in *P. tricornutum*, as reported in the literature. These concentrations were tested to explore nutrient limitations and to identify optimal conditions for maximizing productivity. Additionally, glycerol was included to investigate its role as an external carbon source in promoting biomass and metabolite accumulation. The selection of these nutrients and their concentrations was based on their known effects on algal growth and productivity in previous studies [39, 40].

The light experiment utilized flexible RGB LED strips capable of emitting various colors (blue, red, green, white) with adjustable intensity. Two light levels were tested: low (100 μmol m⁻^2^ s⁻^1^) and high (200 μmol m⁻^2^ s⁻^1^). These intensities were selected to investigate optimal conditions for maximizing biomass and metabolite production, as previous studies have shown that light below 50 μmol m⁻^2^ s⁻^1^ may become limiting for growth and productivity [41, 42]. Light intensity was measured and adjusted using a portable luxometer (Hanna, Mumbai, India) by altering LED quantity and proximity to cultures.

All experiments were conducted in triplicate. Samples were analyzed for optical density, biomass concentration, total lipids, and fucoxanthin content. Cell concentration was determined using UV spectrophotometry (spectrophotometer Hach DR3900) by measuring culture absorbance at 750 nm (OD 750). Sampling was conducted at six time points: 0, 2, 4, 6, 8, and 10 days. Day 0 represents the baseline immediately after inoculation, while days 2, 4, 6, 8, and 10 were chosen to capture key stages in the growth curve.

### Biomass analysis

Biomass was measured by centrifuging 5 mL of culture at 4000 rpm for 10 min, washing the pellet twice with distilled water, and oven-drying the washed biomass at 40 °C until a constant weight was achieved. The dried biomass was then weighed using an analytical balance (± 0.0001 g accuracy) and used to estimate lipid content. All measurements were conducted in triplicate to ensure reliability.

### Lipid extraction and analysis

Total lipids were extracted from the dried biomass following the method of Folch et al. [33] with minor modifications. The dried biomass was suspended in a chloroform/methanol solution (2:1 v/v) and homogenized for 10 min. The mixture was centrifuged at 4000 rpm for 10 min to separate the organic phase containing lipids. The organic phase was collected, and the extraction process was repeated to maximize lipid recovery. The combined organic phases were washed with 0.88% (w/v) KCl solution to remove impurities, and the lipid extract was dried under nitrogen gas. The total lipid content was gravimetrically determined and represented by a percentage of the dry biomass weight.

### Fucoxanthin extraction and analysis

Fucoxanthin was extracted from 5 mL of centrifuged culture biomass that was freeze-dried before extraction. The dried biomass was mixed with 100% ethanol at a ratio of 1:4 mg: mL and incubated at 60 °C for 1 h in darkness to prevent photodegradation of the pigment. After incubation, the mixture was centrifuged at 4000 rpm for 10 min, and the supernatant containing fucoxanthin was collected.

Fucoxanthin content was determined spectrophotometrically using a UV–visible spectrophotometer (Hach DR3900). Absorbance was measured at 445 nm (maximum absorption for fucoxanthin) and 663 nm (to account for chlorophyll interference). Fucoxanthin concentration was calculated using the equation [44]:

1 $$Fucoxanthin \left(mg\, {g}^{-1}DW\right)=\frac{\left[6.39\times \left({A}_{445}\right)-5.18\times \left({A}_{663}\right)\right]}{gDW}.$$

DW is the dry weight of the biomass. All analyses were performed in triplicate.

This approach has been widely used for fucoxanthin quantification in various microalgae species, as it provides a cost-effective and relatively straightforward method for high-throughput analyses. This spectrophotometric method showed a standard error of < 5% when compared to the HPLC results [44].

### Biomass, lipid, and fucoxanthin productivities

Biomass, lipid, and fucoxanthin productivities are typically calculated using data collected over the cultivation period according to Eq. (2):

2 $$Productivity \left( P \right) = \left( {C_{2} - C_{1} } \right) / \left( {t_{2} - t_{1} } \right),$$

where C₂ = final concentration of the content of interest (biomass, lipid, or fucoxanthin) C₁ = initial concentration of the content of interest t₂—t₁ = cultivation time (days).

### GC–MS analysis of *P. tricornutum* fatty acids

Fatty acids were analyzed using a Perkin Elmer Clarus 580/560 S GC–MS system. The oven temperature program started at 80 °C for 6 min., then increased from 8 °C to 280 °C, and held for 4 min. Other parameters included: injection: 280 °C, 1 μL volume, 20:1 split; carrier gas: helium; solvent delay: 5.00 min; transfer temp: 180 °C; source temp: 200 °C; scan range: 50–550 Da and column: Elite-5MS (30 m × 0.25 mm ID, 0.25 μm df).

Fatty acids were identified by comparing retention times and fragmentation patterns with the NIST spectral database. Relative quantities were calculated from the total ion current (TIC) and expressed as normalized peak areas, following [45].

### Evaluation of biodiesel characteristics for the derived algal fatty acid compositions

The evaluation of fuel characteristics for the derived algal fatty acid compositions involved theoretical computations of several biodiesel attributes. These calculations were performed using Eqs. (3) through (8) [46–49]. These properties covered the average degree of unsaturation (ADU %), cetane number (CN), iodine value (IV, g I2.100/g oil), kinematic viscosity (υi, mm2/s), specific gravity (ρ), cloud point (CP, ℃), saponification value (SV, mg KOH/g), long chain saturation factor (LCSF, wt %), higher heating value (HHV), and cold filter plugging point (CFPP, °C).

3 $$\text{Average degree of unsaturation }\left(\text{ADU \%}\right)= \Sigma N \times Mf,$$

where N represents the number of carbon double bonds in fatty acid, and Mf is the mass fraction of each fatty acid.

4 $${\text{Kinematic viscosity }}\left( {{{vi,mm2} \mathord{\left/ {\vphantom {{vi,mm2} s}} \right. \kern-0pt} s}} \right) = \, - {0}{\text{.631 ADU + 5}}{.20,}$$

5 $${\text{Specific gravity }}\left( p \right) = 0.0055 ADU + 0.87,$$

6 $${\text{Cloud point }}\left( {{\text{Cp}},{ }^\circ {\text{C}}} \right) = - 3.35 ADU + 19.99,$$

7 $$\text{Cetane number }(\text{CN}) = -6.67 ADU + 62.87,$$

8 $$\text{Iodine value }\left(\text{IV},\text{ g}\frac{\text{I}2.100}{\text{g}}\text{oil}\right)= 74.37 ADU + 12.71,$$

9 $$\text{Higher heating value }(\text{HHV})= 1.760 \times ADU + 38.53.$$

### Statistical analysis

All data were analyzed in biological triplicates and are presented as mean ± standard deviation (SD). Statistical analysis was performed using a T-test and one-way analysis of variance (ANOVA). The significant differentiation level was set at P < 0.05, compared to the control group.

## Results

### Morphological and molecular identification

Microscopic examination revealed that the isolated strain exhibited the characteristic polymorphic nature of *Phaeodactylum tricornutum*, displaying predominant fusiform cells measuring 25–35 µm in length and 2.5–3.5 µm in width, with pointed ends characteristic of the species. Triradiate cells were observed less frequently. The cell contained a single, central, golden-brown chloroplast typical of diatoms (Fig. S1).

**Fig. 1:**
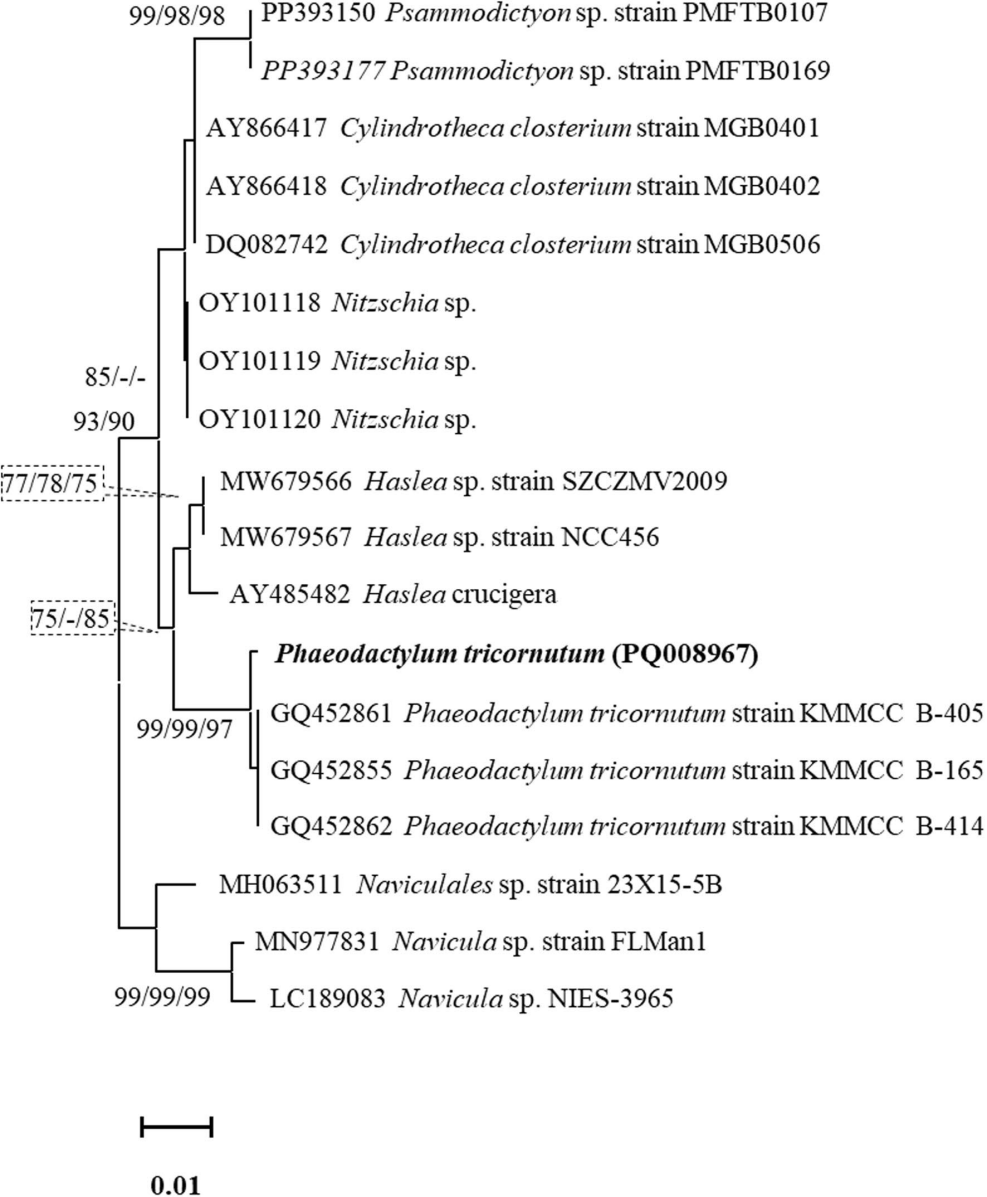
Maximum likelihood dendrograms showing the isolated *Phaeodactylum tricornutum* based on 18S rRNA nucleotide sequences. Bootstrap values greater than 70 are shown on the trees in order of neighbor-joining distance, maximum likelihood, and maximum parsimony (MP) bootstrap, respectively

Figure 1 presents a phylogenetic tree identifying the isolated *Phaeodactylum* sp. and its relation to related taxa, constructed using the ML method based on 18S rRNA gene sequences from 18 strains. Our strain clustered within a distinct clade of *Phaeodactylum* sp. with strong bootstrap support (90%) and showed 95–99% similarity with three specific strains of *P. tricornutum* (KMMCC B-405, B-165, and B-414), confirming its identity as *P. tricornutum*. The sequence data have been deposited in GenBank (accession number PQ008967). This analysis demonstrates the genetic distinctiveness of *P. tricornutum* within related diatom genera and provides robust molecular identification of the species.

### Effect of different concentrations of nutrients on the growth, lipid, and fucoxanthin content of *P. tricornutum*

**Fig. 2:**
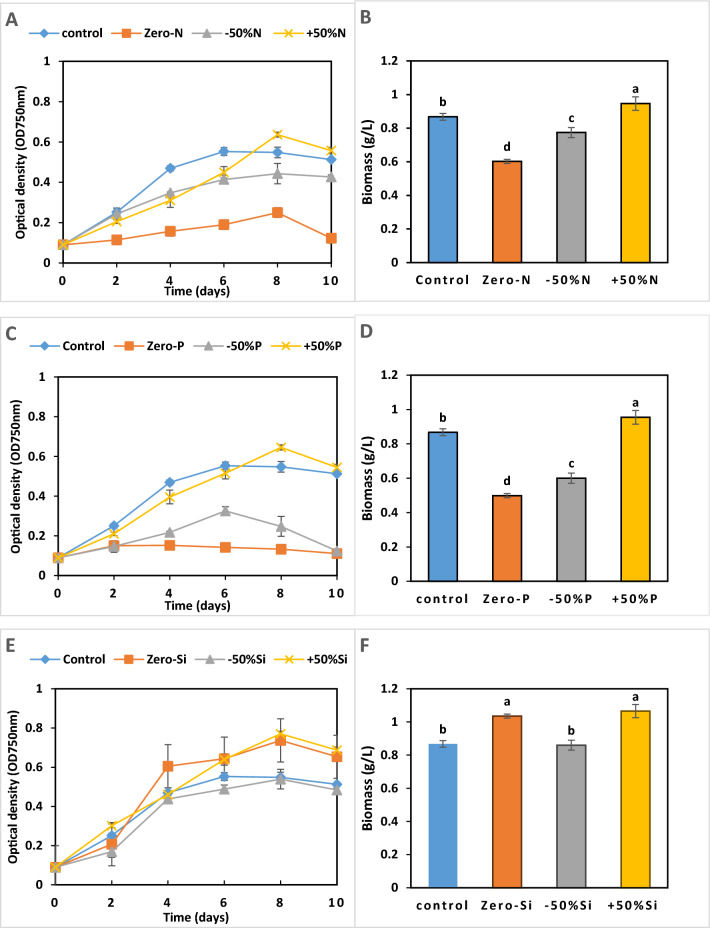
Effect of different concentrations of the major nutrients nitrogen (N) (**A** and **B**), phosphate (P) (**C** and **D**), and silica (Si) (**E** and **F**) on the growth of *Phaeodactylum tricornutum* over 10 days, measured by optical density (OD) and dry weight (DW). The F/2 medium was used as a control. Each value represents the mean of three replicates ± SD. Different lowercase letters above the columns indicate significant differences at *P* < 0.05

The growth of *P. tricornutum* was quantified in media containing various concentrations of nitrogen, phosphorus, and silicate and compared with F/2 media (Fig. 2). All treatments showed an initial 0–2 day lag phase. Nitrogen treatments (Fig. 1A) showed the highest biomass growth for + 50%N (0.96 g L^−1^), peaking at day 8, while Zero-N growth remained stunted (0.5 g L^−1^). While there was a minimum difference between the control and − 50%N. For phosphorus (Fig. 1B), the control, − 50%P, and + 50%P exhibited similar exponential growth from day 2–4, with control and P- + 50 reaching peak biomass (0.95 g L^−1^) at day 8, while − 50%P showed intermediate growth. Zero-P demonstrated severely limited growth. Silicate treatments (Fig. 1C) revealed that all cultures, including Zero-Si, grew rapidly from day 2–6. Zero-Si and + 50%-Si reached similar cell densities, while + 50%Si achieved the highest biomass (1.06 g L^−1^). Most treatments across all nutrients showed a slight decline after day 8, possibly indicating the stationary or death phase onset. Zero concentrations of N, P, and Si significantly reduced *P. tricornutum* growth, while slight increases (+ 50) in these nutrients generally enhanced growth compared to the control.

**Fig. 3:**
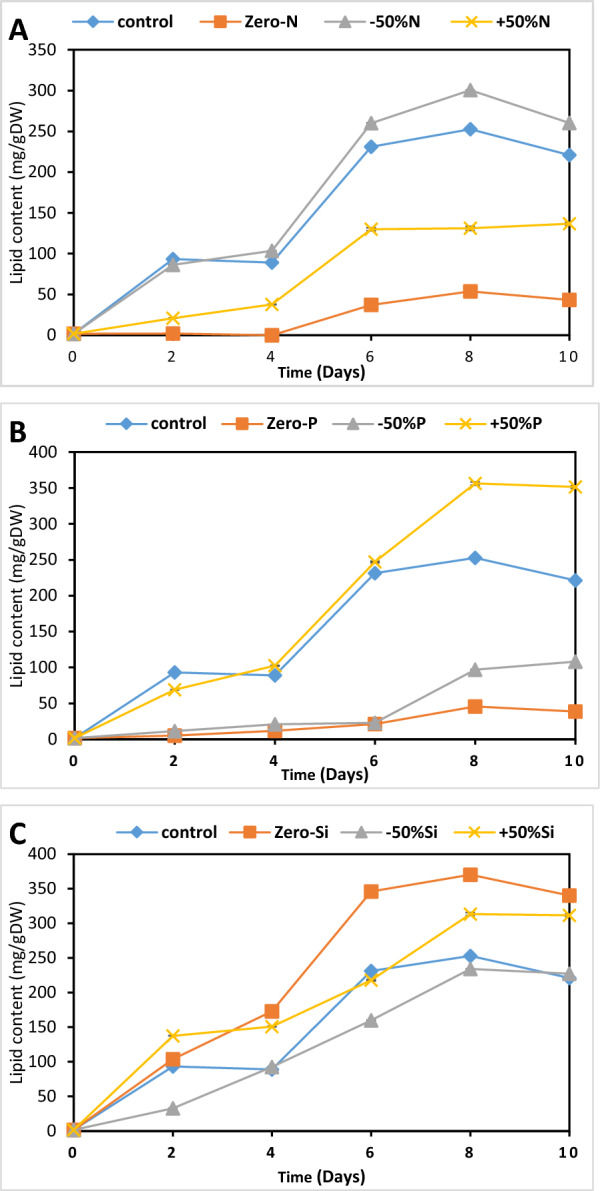
Effect of different concentrations of major nutrients nitrogen (N) (**A**), phosphate (P) (**B**), and silica (Si) (**C**) on the lipid content of *P. tricornutum* for 10 days. F/2 medium was used as a control. Each value is the average of 3 replicates ± SD

The lipid concentration of *P. tricornutum* varied under different nutrient conditions, as shown in Fig. 3. High lipid accumulation was observed in optimized media containing − 50%N, + 50%P, and Zero-Si (277.93, 356.1, and 370. 35 mg g^−1^, respectively) compared to the control F/2 medium (252. 8 mg g^−1^) (Fig. 3). Nitrogen and phosphorus starvation (Zero-N and Zero-P) significantly reduced lipid accumulation (53.5 mg g^−1^ and 45.5 mg g^−1^, respectively) compared to the control (Fig. 3A, B F = 8.59, *P* < 0.001). In contrast, silicate starvation (Zero-Si) produced the maximum lipid content (370.35 mg g^−1^) in *P. tricornutum* compared to the control (Fig. 3C).

**Fig. 4:**
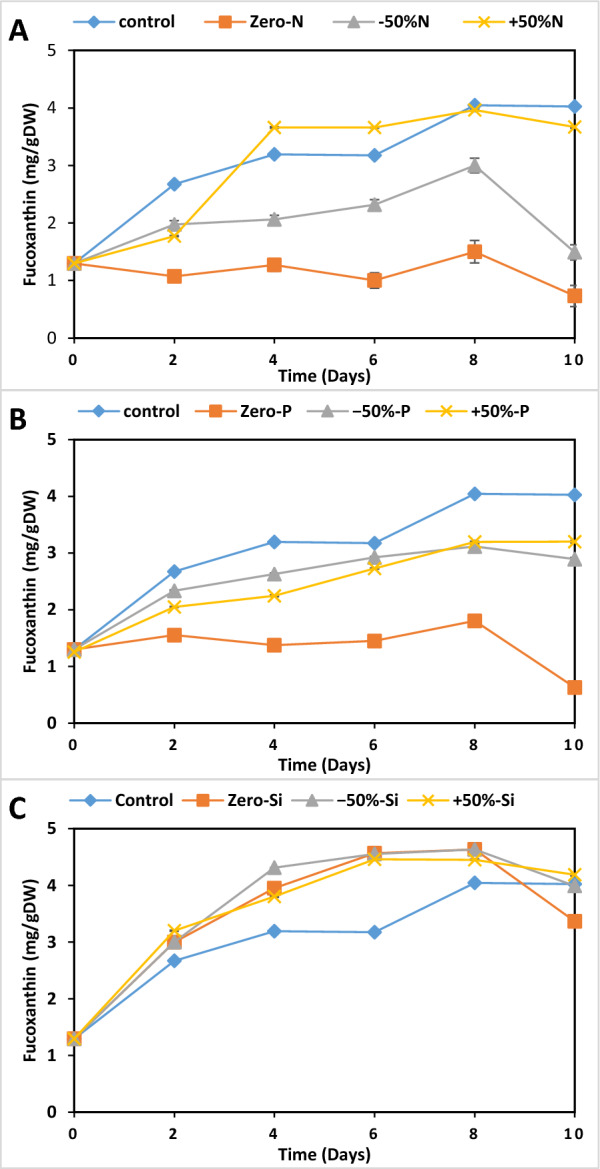
Effect of different concentrations of major nutrients nitrogen (N) (**A**), phosphate (P) (**B**), and silica (Si) (**C**) on the fucoxanthin content of *P. tricornutum* for 10 days under conditions of light, (20 ± 1) °C, 150 rpm. F/2 medium was used as a control. Each value is the average of 3 replicates ± SD

Regarding fucoxanthin (FX) contents, Fig. 4 illustrates the effects of varying concentrations of nitrogen (N), phosphate (P), and silicate (Si) on the fucoxanthin content of *P. tricornutum* over 10 days under controlled light and temperature conditions. Figure 4A (nitrogen variations) reveals that nitrogen availability is crucial for fucoxanthin production. The control, − 50%N, and + 50%N treatments all show similar patterns of increasing fucoxanthin content, reaching about 3.96 mg g^−1^ by day 10. However, in the Zero-N treatment, levels remained below 1.5 mg g^−1^ throughout the experiment.

In Fig. 4B, the control and + 50%P treatments showed similar trends, with fucoxanthin content increasing steadily over time, peaking around day 8–10 at approximately 4.04–4.15 mg g^−1^. The − 50%P treatment follows a similar pattern but with slightly lower values. In stark contrast, the Zero-P treatment significantly reduced fucoxanthin production, remaining below 1.8 mg g^−1^ throughout the experiment.

Figure 4 shows less dramatic differences in fucoxanthin content between treatments. All conditions, including Zero-Si, demonstrate relatively high fucoxanthin content, ranging between 4.04 and 4.63 mg g^−1^ for most of the experiment. The − 50%Si and Zero-Si treatments appear to maintain slightly higher levels (4.63 mg g^−1^), especially in the latter half of the experiment.

To select the best conditions for *P. tricornutum* cultivation, biomass productivity (BP), lipid productivity (LP), and fucoxanthin productivity (FP) for *P. tricornutum* under various nutrient conditions were estimated as shown in Table 1. Our results revealed that silica starvation (Zero-Si) yielded the most favorable outcomes across all three parameters. This condition resulted in high biomass productivity (95.92 mg L^−1^ d^−1^), lipid productivity (49.37 mg L^−1^ d^−1^), and fucoxanthin productivity (0.22 mg L^−1^ d^−1^), outperforming the control and other treatments. Increased phosphorus (+ 50%P) also demonstrated excellent performance, particularly in lipid productivity (60.16 mg L^−1^ d^−1^), while maintaining good biomass (82.42 mg L^−1^ d^−1^) and fucoxanthin production (0.13 mg L^−1^ d^−1^). Interestingly, nitrogen manipulation showed varied effects, with + 50%N exhibiting the best overall performance among nitrogen treatments. While in lipid production, nitrogen starvation showed only 5.15 mg L^−1^ d^−1^, − 50%N showed the highest lipid production of 36.22 mg L^−1^ d^−1^ among nitrate treatments, and moderate biomass production of 62.84 g L^−1^ d^−1^. Conversely, nutrient deprivation, particularly in phosphorus (Zero-P) and nitrogen (Zero-N), significantly impaired all productivity measures. These findings suggest that optimizing silica availability (Zero-Si), followed by phosphorus supplementation (+ 50%P) and nitrogen deficiency (− 50%N), could promote good growth while enhancing lipid production and maintaining fucoxanthin synthesis. Mixotrophic cultivation was performed in the next experiment using glycerol as an organic carbon source for further improvement.

**Table 1:** Biomass productivity (BP), lipid productivity (LP), and fucoxanthin productivity (FP) for *P. tricornutum* under various nutrient conditions on day 8th of cultivation

Productivities	Control	Zero-N	− 50%N	+ 50%N	Zero-P	− 50%P	+ 50%P	Si-zero	− 50%Si	+ 50%Si
BP mg L^−1^ d^−1^	67.93 ± 0.001^c^	23.73 ± 0.00^e^	42.84 ± 0.001^d^	81.08 ± 0.02^b^	6.37 ± 0.02^f^	23.38 ± 0.04^e^	82.42 ± 0.11^b^	95.92 ± 0.01^a^	66.65 ± 0.01^b^	100.97 ± 0.01^a^
LP mg L^−1^ d^−1^	32.62 ± 0.01^d^	5.15 ± 0.01^g^	36.22 ± 0.05^c^	22.46 ± 0.03^e^	4.00 ± 0.11^f^	10.49 ± 0.09^e^	60.16 ± 0.13^a^	49.37 ± 0.14^b^	36.78 ± 0.12^c^	65.48 ± 0.14^a^
FP mg L^−1^ d^−1^	0.20 ± 0.001^c^	0.11 ± 0.001^d^	0.03 ± 0.001^e^	2.19 ± 0.01^a^	0.37 ± 0.001^b^	0.03 ± 0.001^c^	0.13 ± 0.001^d^	0.22 ± 0.002^c^	0.02 ± 0.001^f^	0.04 ± 0.002^e^

The different superscript lowercase letter in each row means a significant difference at *P* < 0.05

### Effect of different concentrations of glycerol on the growth, lipid, and fucoxanthin content of *P. tricornutum*

**Fig. 5:**
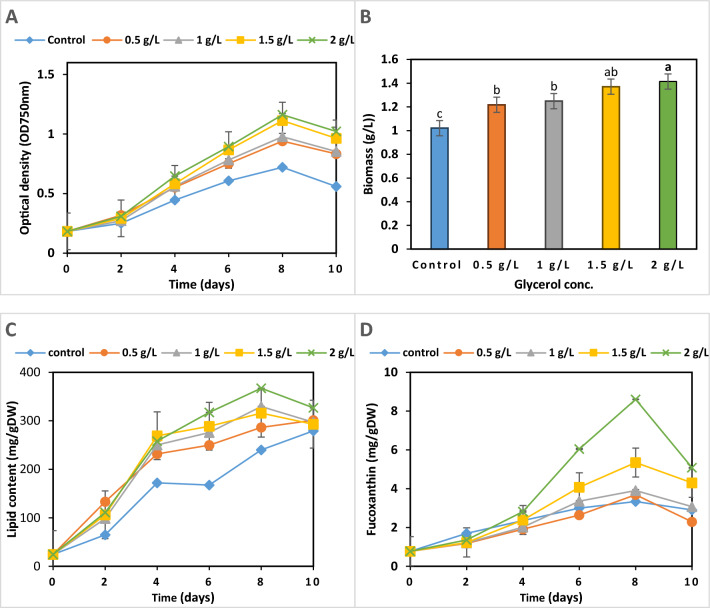
Effect of different glycerol concentrations (0.5, 1, 1.5, and 2 g) on the growth (**A** and **B**), lipid content (**C**), and fucoxanthin content (**D**) of *P. tricornutum* for 10 days under conditions of light, (20 ± 1) °C, 150 rpm. F/2 medium was used as a control. The different lowercase letter above the plotted column means a significant difference at *P* < 0.05. Each value is the average of 3 replicates ± SD

The impact of different glycerol concentrations (0.5, 1, 1.5, and 2 g) on the growth, lipid, and fucoxanthin content of *P. tricornutum* compared to its growth on F/2 medium (control medium) over a 10-day incubation period is illustrated in Fig. 5. Results show that increasing glycerol concentrations generally enhances *P. tricornutum* growth. The 2 g glycerol treatment yields the highest growth, with biomass reaching 1.41 mg L^−1^ by day 8 (Fig. 5A), an increase of about 38% over the control, with biomass productivity reaching 126.53 mg L^−1^ d^−1^.

Regarding lipid accumulation, all treatments have a clear increase in lipid content over time. Data in Fig. 5B show that glycerol supplementation enhances lipid accumulation compared to the control. The 2 g glycerol treatment showed the highest lipid content, reaching approximately 367. 17 mg g^−1^ by day 8 during the late growth phase, with lipid productivity of 89.35 mg L^−1^ d^−1^.

A similar trend was observed in fucoxanthin content. It increased over time for all treatments, with the 2 g glycerol concentration showing the highest yield of 8.60 mg g^−1^ at day 8 compared to the control (Fig. 5C) with fucoxanthin productivity of 0.97 mg L^−1^ d^−1^. Most treatments show a slight decrease in fucoxanthin content from day 8 to day 10.

### Effect of different light regimes on growth, lipid, and fucoxanthin content of *P. tricornutum*

**Fig. 6:**
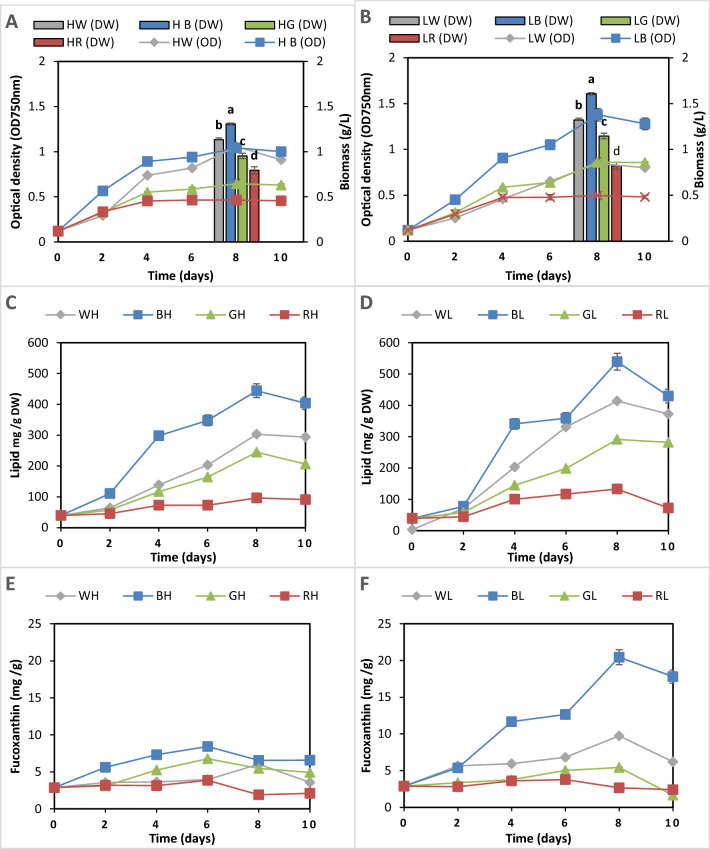
Effect of different light regimes (WH; white high, WL; while low, BH; blue high; BL; blue low; GH; green high; GL; green low; RH; red high and RL; red low) on the growth (**A** and **B**), lipid content (**C** and **D**) and fucoxanthin content (**E** and **F**) of *P. tricornutum* for 10 days. WL using the optimized medium was used as a control. The different lowercase letter above the plotted column means a significant difference at *P* < 0.05. Each value is the average of 3 replicates ± SD

For further improvement, *P. tricornutum* was cultured on the optimized media of − 50%N, + 50%P, Zero silicate, and 2 g glycerol and exposed to four light colors (white, blue, green, red) at two intensities (low = 100 μmol m⁻^2^ s⁻^1^ and high = 200 μmol m⁻^2^ s⁻^1^), as shown in Fig. 6.

Low blue light positively influenced growth at both intensities, with low blue light yielding the highest growth, increasing biomass by 21.2% (to 1.6 g L⁻^1^) at day 8 compared to the optimized medium under standard conditions. Similarly, biomass productivity was also enhanced at the low blue light, resulting in 186.9 mg L^−1^ d^−1^ compared to all different lights followed by HB light (136.76 mg L^−1^ d^−1^), while the lowest biomass productivity was recorded in HR light (51.32 mg L^−1^ d^−1^) (Table 2). This was followed by low white light and high blue light, respectively. Conversely, red light negatively impacted growth, with both low and high intensities resulting in the lowest biomass, decreasing by 37.9% (to 0.82 g L^−1^) and 40.2% (to 0.79 g L⁻^1^), respectively, compared to the control (Fig. 6A and B).

**Table 2:** Biomass productivity (BP), lipid productivity (LP), and fucoxanthin productivity (FP) for *P. tricornutum* under various light regimes on day 8th of cultivation

Productivities	HW	LW	HB	LB	HG	LG	HR	LR
BP mg L^−1^ d^−1^	119.50 ± 2.21^c^	82.37 ± 4.44^e^	136.76 ± 3.61^b^	186.9 ± 3.2^a^	77.92 ± 0.52^f^	109.96 ± 1.8^d^	51.32 ± 1.11^g^	56.12 ± 2.01^g^
LP mg L^−1^ d^−1^	68.55 ± 0.91^b^	47.63 ± 0.8^c^	66.69 ± 0. 5^b^	92.35 ± 0.03^a^	37.36 ± 0.31^d^	46.51 ± 0.69^c^	10.22 ± 0.66^f^	17.70 ± 0.2^e^
FP mg L^−1^ d^−1^	1.15 ± 0.003^c^	0.7 ± 0.002^d^	1.65 ± 0.003^b^	4.2 ± 0.02^a^	1.5 ± 0.001^b^	0.6 ± 0.01^d^	0.3 ± 0.002^e^	0.4 ± 0.001^e^

The different superscript lowercase letter in each row means a significant difference at *P* < 0.05

Lipid content analysis revealed that blue light significantly enhanced lipid production (Fig. 6C and D). LB light resulted in the highest lipid content (539.25 mg g^−1^) at day 8, with lipid productivity of 92.35 mg L^−1^ d^−1^ (Table 2), followed by HB light (443.94 mg g^−1^). Despite its negative effect on growth, red light led to the lowest lipid content at both intensities (132.9 mg g^−1^ for LR, 96.63 mg g^−1^ for HR) compared to the control (Fig. 6C and D).

Fucoxanthin content peaked during the exponential growth phase, reaching maximum levels at day 8 (Fig. 6). Blue light at low intensity had the most significant positive effect on fucoxanthin production (20.44 mg g^−1^) with fucoxanthin productivity of 2.5 mg L^−1^ d^−1^, substantially higher than low-intensity white light (5.4 mg g^−1^). Red light continued to negatively impact fucoxanthin content, with high and low intensities yielding 3.85 and 3.78 mg L^−1^, respectively, on the 6th day (Fig. 6E and F). Interestingly, LW light promoted higher fucoxanthin content (9.73 mg g^−1^) compared to HW light (6.04 mg g^−1^) (Fig. 6E and F).

### Fatty acids profile in *P. tricornutum*

The fatty acid profiles of *P. tricornutum* cultivated under control and optimized conditions showed marked differences (Table 3). The optimized condition resulted in a substantial increase in saturated fatty acids (SFAs), rising from 32.19% in control to 48.72% in the optimized condition. This increase was primarily due to a significant elevation in palmitic acid (C16:0) content, which rose from 24.94% to 39.84%. Conversely, monounsaturated fatty acids (MUFAs) decreased dramatically from 36.12% in control to 20.59% in the optimized condition. Interestingly, oleic acid (C18:1n-9) was only detected in the optimized condition (6.55%). The total polyunsaturated fatty acids (PUFAs) showed a slight increase from 11.68% to 14.71% in the optimized condition, with notable increases in linoleic acid (C18:2n-6) and gamma-linolenic acid (C18:3n-6). However, eicosapentaenoic acid (EPA, C20:5n3) content decreased from 7.04% to 4.80%. The optimized condition also resulted in the appearance of eicosatrienoic acid (C20:3n3) and alpha-linolenic acid (C18:3n3), which were not detected in the control. Overall, the optimized cultivation conditions led to a fatty acid profile dominated by SFAs, potentially improving the oxidative stability of the resulting biodiesel.

**Table 3:** Fatty acid profile (%) of *P. tricornutum* cultivated under optimized conditions (− 50%N, + 50%P, Zero-Si, and 2 g glycerol, low blue light intensity) compared to control, as determined by GC–MS analysis and control using GC–MS mass analysis

Fatty acids	No. of carbon atoms	Fatty acids content (%)	
		Control	Optimized
Myristic acid	C14:0	7.25 ± 0.32	6.27 ± 0.8
Palmitic acid	C16:0	24.94 ± 2.21	39.84 ± 3.61
Stearic acid	C18:0	ND	1.22 ± 0.11
Arachidic acid	C20:0	ND	1.39 ± 0.22
**Total saturated fatty acids (∑SFA)**		**32.19 ± 2.53**	48.72 ± 4.74*
Palmitoleic acid	C16:1t	26.77 ± 3.22	14.04 ± 0.91
cis-10-Heptadecenoic acid	C17:1		ND
Elaidic acid	C18:1t	1.80 ± 0.24	ND
Nervonic acid	C24:1	3.52 ± 0.91	ND
Oleic acid	C18:1(n-9)	ND	6.55 ± 1.01
**Total monounsaturated fatty acids (∑MUFA)**		36.12 ± 5.28*	**20.59 ± 1.01**
Linoleic acid	C18:2(n-6)	2.68 ± 0.6	4.76 ± 0.71
Gamma-linolenic acid (GLA)	C18:3(n-6)	1.96 ± 0.31	3.38 ± 0.11
Eicosapentaenoic acid (EPA)	C20:5n3	7.04 ± 0.41	4.80 ± 0.21
Eicosatrienoic acid	C20:3n3	ND	0.82 ± 0.01
Alpha-linolenic acid(ALA)	C18:3n3	ND	0.93 ± 0.11
**Total polyunsaturated fatty acids (∑PUFA)**		**11.68 ± 1.32**	14.71 ± 1.15*

^*^Significant at *P* < 0.001 using t-test

### Biodiesel properties

Given the high lipid content achieved in the optimized medium and the increased saturated fatty acid content recommended for biodiesel production, we conducted a comparative analysis of biodiesel properties derived from *P. tricornutum* cultivated under control conditions and in the optimized medium. The biodiesel properties from both cultivation conditions were thoroughly analyzed and evaluated against international standards EN 14214 and ASTM D6751 (Table 4). This comparison aimed to assess the impact of our optimization strategy on the quality and suitability of the resulting biodiesel for commercial applications. The optimized condition resulted in a lower average degree of unsaturation (ADU) (0.70 vs 0.79) and iodine value (IV) (64.40 vs 71.12 g I2 100 g^−1^ oil). It also exhibited a higher cetane number (CN) (58.24 vs 57.64). Both conditions produced biodiesel with acceptable kinematic viscosity (KV) (4.71 and 4.77 mm^2^ s^−1^ for optimized and control, respectively) and identical density (0.88 kg L^−1^), meeting ASTM D6751 standards (Table 4). However, the control condition showed a lower cloud point (Cp) (9.51 °C vs 10.71 °C) and slightly higher energy content as indicated by the higher heating value (HHV) (39.92 vs 39.76 MJ kg^−1^).

**Table 4:** Comparison of biodiesel properties from *P. tricornutum* oil cultivated under control and optimized conditions

Property	Control	Optimized	Preferred range	EN 14214	ASTM D6751
Average degree of unsaturation (ADU)	1.03	0.55	Lower is better	NA	NA
Kinematic viscosity (KV) mm^2^/s	4.56	4.86	1.9–6.0 mm^2^/s	3.5–5.0	1.9–6.0
Cetane number (CN)	56.04	59.18	Min. 47	≥ 51	≥ 47
Iodine value (IV) (g I_2_.100 g^−1^ OIL)	88.99	53.96	Max. 120	≤ 120	NA
Cloud point (Cp) (°C)	6.29	12.59	Lower is better	NA	NA
Density (kg L^−1^)	0.88	0.88	0.86–0.90 g/cm^3^	NA	0.86–0.9
Higher heating value (HHV) (Mj kg^−1^)	40.34	39.51	Higher is better	NA	NA

## Discussion

The morphological and molecular analyses provided robust confirmation of the isolate's identity as *Phaeodactylum tricornutum*. The observed microscopic examination of the strain, aligns with previous descriptions of *P. tricornutum* in the literature [16, 50]. The predominance of fusiform cells in our cultures is consistent with findings by Wu et al. [16], who reported that this morphotype typically dominates under standard laboratory conditions. The dimensions of all three morphotypes fell within the ranges previously reported for this species [16].

The molecular identification through 18S rRNA gene sequencing provided additional verification of our morphological observations. The high sequence similarity (95–99%) with established *P. tricornutum* strains in the database, particularly with KMMCC strains, suggests our isolate belongs to a well-characterized clade of this species. The strong bootstrap support (90%) in our phylogenetic analysis further reinforces the reliability of this identification. This molecular confirmation is particularly valuable given that morphological plasticity in *P. tricornutum* can sometimes complicate traditional identification methods.

This study was carried out to determine the impact of various ratios of phosphorus (P), nitrogen (N), and silicate (Si) on the growth, lipid, and fucoxanthin production of the newly isolated *P. tricornutum* for fucoxanthin and renewable biofuels.

Nitrogen is an essential nutrient for algal growth and lipid accumulation, and its concentration strongly influences the growth and metabolic composition of microalgae. Depleting nitrogen in the cultivation medium creates a trade-off: reduced growth is often accompanied by increased lipid productivity [51, 52]. Complete nitrogen depletion, however, severely inhibits algal growth, as observed in this study. Conversely, the addition of 50% more nitrogen (+ 50%N) resulted in the highest biomass production. This indicates that nitrogen limitation, rather than total depletion, is a more effective approach to optimize lipid formation in microalgae [52]**.**

Our results demonstrated that the modified medium containing 50% less nitrogen (− 50%N) produced the highest lipid content, whereas the medium with 50% more nitrogen (+ 50%N) supported the highest biomass growth and productivity. Nitrogen availability plays a crucial role in lipid accumulation, as seen in this study and corroborated by previous findings. While nitrogen starvation is generally associated with enhanced lipid accumulation due to metabolic redirection, our findings highlight the importance of moderate nitrogen limitation. Under − 50%N conditions, lipid accumulation was maximized, while Zero-N conditions resulted in the lowest lipid accumulation. This difference underscores the delicate balance between nitrogen availability and biomass production. Supporting this, Yodsuwan et al. [53] reported that a nitrogen concentration of 475.5 mg/L KNO₃ led to the maximum lipid content (31.14%) in *Chlorella sp.* TISTR 8990 grown in a nitrogen-minimal medium. This suggests that moderate nitrogen limitation, rather than complete nitrogen starvation, is particularly beneficial for lipid production. Similarly, our observation of higher lipid accumulation under − 50%N conditions aligns with findings in *Phaeodactylum tricornutum* Bohlin (CCMP2561), where triacylglycerols (TAGs) increased following nitrate depletion in an f/2 medium containing 0.5 mM NaNO₃ after 60 h of cultivation [54]. This demonstrates that moderate nitrogen stress maximizes lipid accumulation by maintaining metabolic activity and adequate biomass, which are critical for lipid biosynthesis. The low lipid content observed under Zero-N conditions in this study can be attributed to severely reduced biomass due to extreme nitrogen starvation. Nitrogen deprivation halts cell division, redirecting metabolic pathways towards neutral lipid biosynthesis, such as triacylglycerols (TAGs), instead of membrane lipid synthesis [55]. For instance, under nitrogen-limited conditions, *Chlorella* cells achieved a lipid yield of 48.65% of dry weight, which was 62% higher than under nitrogen-replete conditions [51].

Fucoxanthin is crucial as the primary pigment associated with photosynthetic energy transfer to chlorophyll a in diatoms [56]. Beyond its physiological role, fucoxanthin has diverse applications. In healthcare, it demonstrates antioxidant, anti-inflammatory, and anticancer properties, with potential for treating obesity and metabolic disorders. In cosmetics, it protects against UV damage and supports anti-aging formulations [57]. In nutraceuticals, fucoxanthin enhances mitochondrial function and energy metabolism, making it a valuable dietary supplement. It also holds promise in agriculture and aquaculture, improving crop resilience and pigmentation in farmed species. Sustainable production from diatoms like *Phaeodactylum tricornutum* is vital for expanding its industrial use [58].

Sufficient nitrogen is essential for biomass production and fucoxanthin accumulation [12, 15]. Meanwhile, the depletion of nitrogen is detrimental to the synthesis of fucoxanthin. It has been found that in nitrogen-depleted *P. tricornutum* cultures, the contents of fucoxanthin, chlorophyll a, and chlorophyll c decreased [59]. According to Frick et al. [42], all three investigated *P. tricornutum* strains showed a decrease in fucoxanthin content during nitrogen depletion. This decrease may be explained by the suppression of genes involved in photosynthesis and chlorophyll under low nitrogen levels, which also prevents the production of novel pigments, including nitrogen-free pigments like fucoxanthin [61, 62]. Our findings, which showed a decrease in fucoxanthin content with nitrogen depletion (− 50%N and Zero-N, respectively), are consistent.

Phosphate is necessary for nucleic acids, phospholipids, and intermediate metabolites [63]. Our study demonstrated that increased phosphate concentration (+ 50) significantly enhanced growth, lipid content, fucoxanthin and compared to phosphate-deficient conditions. These results are consistent with Guo et al. [64] who demonstrated that in *Scenedesmus obliquus*, phosphate supplementation resulted in higher algal biomass (9.9 g L^−1^) and lipid content (49.2%) when compared to cultures lacking phosphate supplements. They postulated that elevated levels of orthophosphate aided in the creation of lipids, pigment, and vegetative development by promoting the conversion of inorganic phosphate to organic phosphate and quickening phosphate intake. Chu et al. [65], found that *C. vulgaris*’s lipid productivity for biodiesel synthesis was 58.39 mg L^−1^d^−1^, exceeding phosphate-deficient and control conditions when grown in phosphate-sufficient circumstances. They also noticed that, compared to the control medium, the phosphate uptake rate of *C. vulgaris* rose 3.8 times. Conversely, phosphate limitation can negatively impact microalgal growth and metabolism. Under phosphate-limited conditions, carbon partitioning is disrupted, preventing efficient CO_2_ utilization and starch synthesis and reducing growth [66]**.** Alipanah et al. [47] stated phosphate restriction in *P. tricornutum* decreased cell density and chlorophyll a content.

Regarding the effect of phosphate on fucoxanthin, our results showed no significant difference in fucoxanthin content between + 50% and − 50% phosphate concentrations relative to the standard F/2 medium. This minimal effect may be attributed to the relatively low phosphate requirements for *P. tricornutum*. Sun et al. [48] found that phosphate concentration had a less pronounced effect than nitrogen concentration on the growth and fucoxanthin content of the diatom *Nitzschia* sp. Specifically, increasing phosphate three times from 1.13 to 4.5 mg L⁻^1^ resulted in only a 1.1-fold increase in fucoxanthin, while an increase in nitrogen 1.3 times from 75 to 100 mgL^−1^ led to a 1.7-fold increase in fucoxanthin. Moreover, a high residue of phosphate left at the end of the culture at 4.5 mgL^−1^ phosphate concentration was not observed at lower phosphate concentrations. Similarly, Marella and Tiwari [49] found that the greatest cell growth and fucoxanthin content were obtained at a 10 mg L⁻^1^ phosphate concentration but with no significant differences between 10 mg L⁻^1^ and 20 mg L⁻^1^ phosphate concentrations. These findings collectively suggest that while phosphate is essential for microalgal growth and fucoxanthin production, its impact may be less dramatic than nitrogen’s, particularly at concentrations above a certain threshold.

Silicate is a crucial nutrient for diatoms, as it is used to build their cell walls [70]. Surprisingly, both silicate concentrations (Zero-Si and Si + 50) had almost similar results for growth in the obtained results, but lipid content partly varied with previous concentrations. This unexpected outcome can be attributed to the unique characteristics of *P. tricornutum*. This species is known for its low silicate requirements and metabolic plasticity [71]. It can produce both silicified (oval form) and non-silicified (fusiform and triradiate forms) organic cell wall morphotypes, allowing it to thrive even under silicate-limited conditions [71, 72]. Moreover, *P. tricornutum* has only one valve of its oval morphotype, which contains silicon, and its frustule is only lightly silicified [73]. The unique characteristic of minimal silicification in *P. tricornutum*, particularly notable in our observations, represents an unusual trait among diatoms and may contribute to its widespread use as a model organism in biological studies [74]. This reduced silica requirement might also have important implications for biotechnology applications, as it potentially reduces cultivation costs compared to heavily silicified diatom species.

Interestingly, while growth rates were similar, lipid content varied between the two conditions. An increase of 15.39% in lipid contents was recorded for zero-Si compared to + 50%Si. This differential lipid accumulation aligns with the established understanding that nutrient stress, including silicate limitation, often triggers increased lipid production in microalgae [75]. The increased lipid content observed under silicate limitation can be attributed to a metabolic shift. When silicate is scarce, *P. tricornutum* redirects carbon flux from silica deposition towards lipid biosynthesis, particularly triacylglycerols (TAGs) [76]. This metabolic plasticity allows *P. tricornutum* to maintain growth while simultaneously accumulating lipids under silicon-limited conditions. These findings highlight the complex interplay between nutrient availability, growth, and lipid production in *P. tricornutum*. They also underscore the importance of considering multiple factors when interpreting results related to nutrient manipulation in microalgal cultivation.

Our study revealed a surprising ability of *P. tricornutum* to produce relatively high fucoxanthin content across all silicate concentrations, including zero-silicate media. Notably, the highest fucoxanthin production (4.63 mg g^−1^) was detected in the zero-silicate condition, followed by + 50%Si with no significant differences. This finding suggests that fucoxanthin biosynthesis in *P. tricornutum* may be independent of silicate concentration in the F/2 medium that contains 30 mg/L. Recent studies have explored the effects of nutrient concentrations on fucoxanthin production in various microalgae species. High silicate concentrations were found to enhance fucoxanthin synthesis in *Nitzschia laevis* under heterotrophic conditions [77], while high silicate concentration did not affect fucoxanthin accumulation in the diatom *P. tricornutum* [78]. However, the impact of silicate on fucoxanthin production seems to be species-specific, as it was not mentioned as a significant factor for *Isochrysis galbana* [79].

Overall, this data suggests that while *P. tricornutum* can maintain fucoxanthin production under various nutrient conditions, phosphorus and nitrogen are essential for optimal synthesis. Silicate appears less critical, as even Zero-Si conditions allow for substantial fucoxanthin production. These findings have implications for optimizing culture conditions to enhance fucoxanthin yields in *P. tricornutum*.

For further biomass, lipid, and fucoxanthin improvement, glycerol was utilized as the sole organic carbon source in the mixotrophic condition of *P. tricornutum.* The current results demonstrate that glycerol positively influences microalgal growth, lipid content, and fucoxanthin production. These findings align with Choi and Yu [39], who reported that biomass and lipid concentrations in *C. vulgaris* and *Botryococcus braunii* improved significantly when grown in a medium containing 5 g L^−1^glycerol, with increases in lipid content of 39.42% and 57.82%, respectively, compared to autotrophic conditions. Rai and Gupta [40] found that 1% glycerol (v/v) doubled the growth of *Scenedesmus* sp. compared to photoautotrophic cultivation, while 5% glycerol resulted in maximum lipid accumulation of 52.32% compared to 12.55% in control. These studies consistently show that glycerol enhances both biomass and lipid content in microalgae, making it a promising substrate for improving biofuel production efficiency.

As observed in this study, glycerol stimulates fucoxanthin production at all tested glycerol concentrations, resulting in a 2.5% increase compared to the control without glycerol. In this context, mixotrophic cultivation using glycerol has increased biomass and fucoxanthin by 37.76% and 26.86%, respectively, in *P. tricornutum* [80]. The addition of glycerol (2 g L⁻^1^) to the growth medium of *Cylindrotheca* sp. resulted in a 52% increase in biomass and a 29% increase in fucoxanthin yields compared to the autotrophic culture (control) without negatively affecting photosynthetic performance [81].

For further improving biomass lipid and fucoxanthin content optimized medium consists of F/2 medium with slightly reduced nitrogen (− 50%N), increased phosphorus (+ 50%P), no additional silicate (Si-zero), and 2 g L^−1^ of glycerol supplementation were subjected to different light regimes. Light intensity plays a crucial role in modulating microalgal cellular composition and physiological responses [12, 82]. Under low-light conditions, microalgae typically enhance their photosynthetic efficiency by increasing the concentration of light-harvesting pigments, including chlorophyll and primary carotenoids. Conversely, high light exposure triggers a reduction in these light-capturing pigments while simultaneously stimulating the production of photoprotective compounds such as ß-carotene [82]. These adaptive responses reflect the sophisticated photoacclimation mechanisms that enable microalgae to optimize their photosynthetic performance across varying light conditions. Red, blue, and green light are algae’s primary regulators of carbon flow distribution [83]. Blue light exposure in various algal species increases chlorophyll content, glycolate production, amino acid concentrations, and TCA cycle intermediates, while red light promotes amino acid production and carbohydrate accumulation [84, 85]. Blue light perception by phototropin inhibits starch accumulation in *Chlamydomonas* [86]. Blue light can trigger enzymatic activation and control gene expressions linked to photoprotective reactions [87]. Compared to other light colors, blue light significantly increases lipid content during the exponential growth phase [88]. These findings highlight the intricate relationship between light quality and carbon metabolism in algae, demonstrating rapid metabolic reorganization in response to changing light conditions. The present study showed a similar pattern: BL, especially at low light intensities of 100 μmol m^−2^ s^−1^, improved biomass by 21.2% and lipid content by 30% compared to LW rather than having a detrimental effect on development, in addition to light color and intensity effects on pigments. Diatoms show higher functional absorption of blue and red light by photosystem II compared to white light, leading to more efficient photochemical energy conversion and enhanced non-photochemical quenching [89]. Optimal combinations of blue and red light, as well as specific intensities and photoperiods, have been shown to enhance growth, biomass, lipid content, and pigment production in various algal species [90, 91].

Switching from white to blue LED lighting during mixotrophic cultivation can significantly boost fucoxanthin productivity in diatoms [80]. These results highlight the potential of mixotrophic cultivation and LED color changes as promising strategies for enhancing fucoxanthin productivity in diatoms. Similar results were obtained in the present study, where fucoxanthin productivity improved from 0.7 mg l^−1^ d^−1^ at LW to 4.2 mg L^−1^ d^−1^ at LB under mixotrophic cultivation using 2 g L^−1^ glycerol. The change of the diadinoxanthin cycle, where diatom cells tend to collect more diatoxanthin instead of fucoxanthin to counter photoinhibition under greater light intensity, might be related to the positive effects of blue light at lower light intensity on fucoxanthin productivity [92]. For instance, in autotrophic mode, blue light (BL) may increase the accumulation of fucoxanthin while red light may encourage cell development [90, 93].

Medium–high light intensity was shown to support maximal growth in *Fistulifera solaris*. However, low light intensity produced the highest fucoxanthin content of 11.16 mg L^−1^, while high intensity only produced 3.36 mg L^−1^ [94]. Under continuous light, *Isochrysis zhangjiangensis* showed maximal fucoxanthin productivity of 100 μmol m^−2^ s^−1^. An ideal balance between biomass accumulation and fucoxanthin synthesis was achieved by this light intensity [95]. However, growth phase and irradiance can greatly impact the pigment ratios in various phytoplankton species. In *Cylindrotheca closterium*, fucoxanthin/chlorophyll a ratios declined with irradiance, although most marker pigments to chlorophyll a ratios were independent of growing irradiance [95]. In *P. tricornutum,* the highest values of fucoxanthin content (1.7 mg g^−1^) achieved under low-light conditions were significantly higher than those obtained under high-light conditions of only 0.54 mg g^−1^ [96].

Nutrient concentration and light intensity are critical factors that influence the fatty acid profile of microalgae. These environmental parameters can be adjusted to optimize lipid production and tailor the fatty acid composition for specific applications, such as biodiesel production [52]. Nutrient manipulation, especially variations in nitrogen and phosphorus levels, has been shown to significantly impact lipid accumulation and fatty acid profiles. For example, Sharma et al. [75] demonstrated that nitrogen limitation in microalgae often leads to an increase in total lipid content, particularly saturated and monounsaturated fatty acids.

In the present study, under control conditions, *P. tricornutum* exhibited the highest levels of SFA, followed by MUFA and PUFA. This pattern was consistent with various studies (Table 5). Yodsuwan et al. [17], reported that nitrate deficiency alters the fatty acid profile of *P. tricornutum*, enhancing SFA content, particularly palmitic acid, while palmitoleic acid was identified as the sole unsaturated fatty acid. The observed increase in SFAs in our study aligns with these findings, as nitrogen limitation often triggers enhanced synthesis of storage lipids such as triacylglycerols (TAGs), which are rich in SFAs [98]. This shift is advantageous for biodiesel production, as higher SFA content enhances oxidative stability.

**Table 5:** Comparison of biomass production, lipid yield, fucoxanthin levels, and fatty acid composition of *P. tricornutum* with findings from related studies in the literature

Strain	Culture medium	Light intensity (µmol photons m⁻^2^ s⁻^1^)	biomass (g/L)	Total lipid content (% DW)	SFA (%)	MUFA (%)	PUFA (%)	Dominant FA	Fucoxanthin (mg/g DW)	Ref
*P. tricornutum* FACHB-863	700-mL photobioreactor. Flask F/2	100	0.81	–	–	–	–	–	13	[13]
*P. tricornutum* FACHB-863	700-ml photobioreactor. SW:water = 1:1	100	1.54	–	–	–	–	–	21.2	[13]
*P. tricornutum*	20-L polycarbonate flasks	200	0.96		26.52	21.91	32.02	C20:5	–	[14]
*P. tricornutum* FACHB-863	200-mL flask F/2-Si	150	0.55	20	47.85	28.66	24.57	C16:0	–	[50]
*P. tricornutum* SCSIO140 SCSIO771, SCSIO431, SCSIO433, and SCSIO766	2-L F/2-Si	40	0.36	22.5	34.7		17.48	C16:0	3.7	[16]
*P. tricornutum* SCSIO771	2-L F/2 -Si	40	0.28	30.75	34.19		15.52	C16:0	3.58	[16]
*P. tricornutum* SCSIO431	2-L F/2 -Si	40	0.33	23	38.45		18.26	C16:0	2.14	[16]
*P. tricornutum* SCSIO433	2-L F/2 -Si	40	0.28	15.91	44.23		19.2	C16:0	2.23	[16]
*P. tricornutum* SCSIO766	2-L F/2 -Si	40	0.29	24	25.82		27.35	C16:0	4.35	[16]
*P. tricornutum* SCSIO828	2-L F/2 -Si	40	0.24	24.5	22.61		34.29	C16:0	5.5	[16]
*P. tricornutum*	350-mL flask F/2-Si	100	0.86	25.28	32.19	36.12	11.68	C16:0	4.04	This study
	350-mL flask optimized F/2	100	1.6	53.9	48.72	20.59	14.71*	C16:0	20.44	This study

Light intensity also plays a crucial role in modifying fatty acid profiles. It was observed that *Chaetoceros calcitrans* accumulated more saturated fatty acids under high light intensities, while low-light conditions promoted the synthesis of polyunsaturated fatty acids [99]. This finding aligned with this study, which showed an increase in PUFA content using an LB-optimized culture compared to the control of a normal F/2 medium for LW. Another study confirms present results; Duarte et al. [77] reported that shorter wavelengths in the blue spectral range resulted in a higher yield of total fatty acids, especially SFA in *P. tricornutum*.

Additionally, it was discovered that blue LED produced the most biomass while green LED produced the highest lipid content [101]. The effectiveness of blue light in promoting lipid accumulation, coupled with its energy efficiency, makes it an attractive option for industrial applications. It investigated the energy consumption of different light sources for microalgal cultivation. They found that blue LED light could reduce energy input by approximately 50% compared to red-blue LED combinations and by about 75% compared to white fluorescent light while still maintaining high biomass productivity [93].

In our study, the optimized conditions, including modifications in nitrogen and phosphorus levels and low-intensity blue light, resulted in a marked increase in saturated fatty acids, particularly palmitic acid. This aligns with previous findings and suggests that our optimization strategy effectively modulated the fatty acid profile for improved biodiesel properties.

These findings highlight the potential of using environmental factors to tailor the fatty acid composition of algae and diatoms for specific applications, offering a promising approach for enhancing biofuel feedstock quality.

To assess the biodiesel quality under these conditions, we analyzed several key properties crucial for engine performance, fuel stability, and environmental impact. The length of the carbon chain, cetane number (CN), and degree of unsaturation (DU) in the fatty acid profile are significant factors affecting biodiesel quality, such as oxidative stability and low-temperature properties [102]. The optimized culture condition under BL of higher content of saturated FA (48.72%) resulting in lower ADU is generally better as it indicates higher oxidative stability [103]. Both cultures are within the acceptable range of the Kinematic Viscosity [104, 105]. The fatty acids generated at optimized conditions have a higher CN (58.24 vs 57.64), indicating better ignition quality and potentially lower emissions [106]. The optimized condition has a much lower IV, suggesting better oxidative stability. The optimized condition has slight differences in cloud point compared to the control, making it suitable for cold weather. Both conditions have the same density (0.88), within the acceptable range. The control has a slightly higher HHV (40.34 vs 39.51), indicating a marginally better energy content. While both conditions produce biodiesel with acceptable properties, the optimized condition appears to be superior overall. It shows significant improvements in cetane number and iodine value, crucial for fuel quality and storage stability. The lower ADU also supports better oxidative stability.

The synergistic effects of nutrient limitation, optimized light intensity, and glycerol addition improved lipid content and maintained substantial fucoxanthin levels, highlighting *P. tricornutum*’s potential as a versatile feedstock for biorefinery applications. These results contribute to the growing knowledge of diatom cultivation and open new avenues for optimizing microalgal-based production systems.

## Conclusion

In conclusion, this study successfully optimized cultivation parameters for *Phaeodactylum tricornutum*, enhancing biomass, fucoxanthin pigment, lipid production, and improving key biodiesel properties. The optimized conditions, including nutrient modifications (− 50%N, + 50%P Zero-Si, and addition of 2 g glycerol) and low-intensity blue light, significantly improved the biomass, fucoxanthin, compared to the control or single factor experiment. Also, increasing FA content to the maximum altered the fatty acid profile, increasing saturated fatty acids from 32.19% to 78.73%. This improved biodiesel characteristics, such as higher cetane number and lower iodine value, indicating better combustion quality and oxidative stability. However, these improvements came with trade-offs in cloud point and heating value. *P. tricornutum* remarkably adapted to varied nutritional conditions, highlighting its potential as a versatile biofuel feedstock. Its ability to significantly modify lipid content and composition in response to environmental changes makes it a promising candidate for biodiesel production. Future research should focus on balancing these properties for optimal performance, scaling up production, and conducting comprehensive sustainability assessments to establish *P. tricornutum* as a viable, sustainable biodiesel source.

## Supplementary Information

Additional file 1

## Data Availability

No datasets were generated or analyzed during the current study.
