# Supplementary material for: Optimizing Phaeodactylum tricornutum cultivation: integrated strategies for enhancing biomass, lipid, and fucoxanthin production
Source: Biotechnol Biofuels Bioprod. 2025 Jan 18;18:7. doi: 10.1186/s13068-024-02602-5 (PMC11742496; doi:10.1186/s13068-024-02602-5)
Supplement: Supplementary file 1 — Additional file 1 [file 13068_2024_2602_MOESM1_ESM.docx]

**Optimizing Phaeodactylum tricornutum Cultivation: Integrated Strategies for Enhancing Biomass, Lipid, and Fucoxanthin Production**

Mostafa Elshobary^1,2^^[[1]](#footnote-1)^, Walaa A. Abo-Shanab^1^, Stephan Ende^2^, Mohammed Alquraishi^3^, Rania A. El-Shenody^1^

^1^ Botany and Microbiology Department, Faculty of Science, Tanta University, 31527 Tanta, Egypt.

^2^ Aquaculture Research, AWI – Helmholtz Centre for Polar and Marine Research, Am Handelshafen, 27570 Bremerhaven, Germany

^3^ Department of Community Health Sciences, College of Applied Medical Sciences, King Saud University, 11421 Riyadh, Saudi Arabia


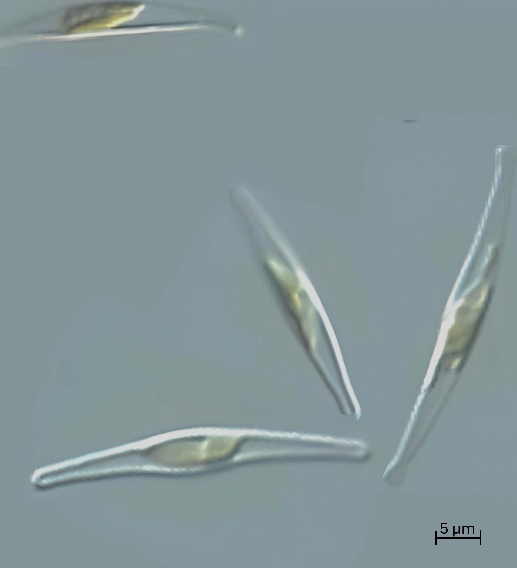


Fig.S1. Light microscopic images of cell morphology of the isolated *P. tricornutum*, Scale bars: 5 μm.

**Table S1**. F/2 medium composition

| Nutrients | concentrations |
| --- | --- |
| NaNO_3_ | 880 μM |
| NaH_2_PO_4_ | 36 μM |
| Na_2_SiO_3_ | 140 μM |
| FeCl_3_ | 12 μM |
| CuSO_4_ | 41 μM |
| ZnSO_4_ | 76 μM |
| MnSO_4_ | 940 μM |
| Na_2_MoO_4_ | 37 μM |
| CoCl_2_ | 37 μM |
| vitamins | (Thiamin HCl (200 mg L⁻¹), Cyanocobalamin (10 mg L⁻¹) and Biotin (100 mg L⁻¹) |

**Table S2** Different Exp.s of optimized media consists of different concentrations of major nutrients [Nitrogen (N): Zero-N, −50%N%, +50%N; Phosphorus (P): Zero-P, −50%P, P+50%P; Silicate (Si): Si-Zero, −50%Si, +50%Si and glycerol concentration of (0.5, 1, 1.5, and 2 g L⁻1)], different glycerol concentrations (0.5ml, 1ml, 1.5ml and 2ml) and diﬀerent light regimes (WH; white high, WL; while low, BH; blue high; BL; blue low; GH; green high; GL; green low; RH; red high and RL; red low) compared with control F/2 media for cultivation of *P. tricornutum*

|  | **Factors** | | | |
| --- | --- | --- | --- | --- |
| **Experiments** | **Nitrate** | **Phosphate** | **Silicate** | **Glycerol (g)** |
| Control Exp. 1 | 880 μM | 36 μM | 140 μM | - |
| Exp. 2 | **+50 (1320 μM)** | 36 μM | 140 μM | - |
| Exp. 3 | **-50 (440 μM)** | 36 μM | 140 μM | - |
| Exp. 4 | **Zero** | 36 μM | 140 μM | - |
| Exp. 5 | 880 μM | **+50 (54 μM)** | 140 μM | - |
| Exp. 6 | 880 μM | **-50 (18 μM)** | 140 μM | - |
| Exp. 7 | 880 μM | **Zero** | 140 μM | - |
| Exp. 8 | 880 μM | 36 μM | **+50 (210 μM)** | - |
| Exp. 9 | 880 μM | 36 μM | **-50 (70 μM)** | - |
| Exp. 10 | 880 μM | 36 μM | **Zero** | - |
| Exp. 11 | 880 μM | 36 μM | 140 μM | **0.5** |
| Exp. 12 | 880 μM | 36 μM | 140 μM | **1** |
| Exp. 13 | 880 μM | 36 μM | 140 μM | **1.5** |
| Exp. 14 | 880 μM | 36 μM | 140 μM | **2** |

1. Correspondance:

   [mostafa.elshobary@awi.de](mailto:mostafa.elshobary@awi.de); [mostafa_elshobary@science.tanta.edu.eg](mailto:mostafa_elshobary@science.tanta.edu.eg) (M. Elshobary)

   Aquaculture Research, AWI – Helmholtz Centre for Polar and Marine Research, Am Handelshafen, 27570 Bremerhaven, Germany [↑](#footnote-ref-1)
